# Divergent functions of the Arabidopsis mitochondrial SCO proteins: HCC1 is essential for COX activity while HCC2 is involved in the UV-B stress response

**DOI:** 10.3389/fpls.2014.00087

**Published:** 2014-03-25

**Authors:** Iris Steinebrunner, Uta Gey, Manuela Andres, Lucila Garcia, Daniel H. Gonzalez

**Affiliations:** ^1^Department of Biology, Technische Universität DresdenDresden, Germany; ^2^Instituto de Agrobiotecnología del Litoral (CONICET-UNL), Universidad Nacional del LitoralSanta Fe, Argentina

**Keywords:** SCO (synthesis of cytochrome *c* oxidase), mitochondria, copper chaperone, COX complex, UV-B stress, plant growth and development, BN-PAGE, *Arabidopsis thaliana*

## Abstract

The two related putative cytochrome *c* oxidase (COX) assembly factors HCC1 and HCC2 from *Arabidopsis thaliana* are Homologs of the yeast Copper Chaperones Sco1p and Sco2p. The *hcc1* null mutation was previously shown to be embryo lethal while the disruption of the *HCC2* gene function had no obvious effect on plant development, but increased the expression of stress-responsive genes. Both HCC1 and HCC2 contain a thioredoxin domain, but only HCC1 carries a Cu-binding motif also found in Sco1p and Sco2p. In order to investigate the physiological implications suggested by this difference, various *hcc1* and *hcc2* mutants were generated and analyzed. The lethality of the *hcc1* knockout mutation was rescued by complementation with the *HCC1* gene under the control of the embryo-specific promoter *ABSCISIC ACID INSENSITIVE 3*. However, the complemented seedlings did not grow into mature plants, underscoring the general importance of *HCC1* for plant growth. The HCC2 homolog was shown to localize to mitochondria like HCC1, yet the function of *HCC2* is evidently different, because two *hcc2* knockout lines developed normally and exhibited only mild growth suppression compared with the wild type (WT). However, *hcc2* knockouts were more sensitive to UV-B treatment than the WT. Complementation of the *hcc2* knockout with *HCC2* rescued the UV-B-sensitive phenotype. In agreement with this, exposure of wild-type plants to UV-B led to an increase of *HCC2* transcripts. In order to corroborate a function of HCC1 and HCC2 in COX biogenesis, COX activity of *hcc1* and *hcc2* mutants was compared. While the loss of *HCC2* function had no significant effect on COX activity, the disruption of one *HCC1* gene copy was enough to suppress respiration by more than half compared with the WT. Therefore, we conclude that HCC1 is essential for COX function, most likely by delivering Cu to the catalytic center. HCC2, on the other hand, seems to be involved directly or indirectly in UV-B-stress responses.

## Introduction

Mitochondrial (mt) biogenesis requires the assembly of proteins synthesized in two different compartments. In addition, due to the essential redox nature of many processes that take place in mitochondria, the formation of active components also requires the synthesis, transport and insertion of a set of co-factors (Herrmann and Funes, 2005; Barrientos et al., 2009; Kim et al., 2012). Among these, copper is one of the components which is required for the activity of cytochrome *c* oxidase (COX or complex IV; Cobine et al., 2006). COX contains two copper centers located in the subunits COX1 and COX2. Insertion of copper into COX is an intricate process that requires the participation of several mt proteins that function in either its delivery or redox reactions related with the assembly process (Herrmann and Funes, 2005; Cobine et al., 2006). The occurrence of COX assembly factors in prokaryotes suggests that some of them were already present in the endosymbiont that originated the mitochondrion while others are more recent acquisitions.

A family of proteins that has been related with copper insertion into COX, particularly into the COX2 subunit, is the SCO family. SCO proteins are of prokaryotic origin and usually contain a transmembrane domain and a soluble domain that contains redox-active cysteines and a histidine presumably involved in copper binding (Banci et al., 2011). The fact that the soluble domain contains a thioredoxin fold has prompted some authors to postulate that SCO proteins do not act in the direct transfer of copper to COX but rather in the reduction of the COX2 cysteines involved in copper binding (Balatri et al., 2003; Abriata et al., 2008). SCO proteins were first analyzed in *Saccharomyces (S.) cerevisiae* mutants defective in COX assembly (hence their name, Synthesis of Cytochrome *c* Oxidase; Schulze and Rödel, 1988). However, current evidence of the presence of SCO proteins in bacteria that do not contain COX-like proteins (Arnesano et al., 2005; Banci et al., 2007) suggests that some members of the family may have different or additional functions. In support of this, *S. cerevisiae* contains two *SCO* genes, but only *SCO1* is essential for COX assembly, while mutations in *SCO2* do not have a significant effect (Glerum et al., 1996).

Also higher eukaryotes like humans and seed plants contain more than one *SCO* gene. However, the duplication events that led to this divergence seem to have occurred independently (Attallah et al., 2011). Accordingly, the functional consequences of duplication also seem to differ. Unlike the case in *S. cerevisiae* mentioned above, both human SCO proteins participate in COX assembly, but they are not redundant and fulfill different roles (Leary et al., 2004). In plants, knockout (KO) mutations of the *Arabidopsis (A.) thaliana HCC1* gene caused embryo lethality, possibly due to defects in COX assembly (Attallah et al., 2011; Steinebrunner et al., 2011). This hypothesis is supported by its localization in mitochondria (Steinebrunner et al., 2011) and the presence of a Cu-binding motif. A *HCC2* mutation altered the expression of genes related to copper homeostasis and stress responses, but contrary to the KO of *HCC1*, did not show a strong phenotype change compared with the wild type (WT) under normal growth conditions (Attallah et al., 2011).

In the present work, we reinforce the argument that the two proteins have divergent functions. Mutants with only one intact *HCC1* copy showed diminished COX activity, corroborating that HCC1 is indeed required for complex IV assembly. The *HCC2* loss-of-function, on the other hand, did not impair COX activity, but reduced the tolerance to UV-B stress. We summarize our data in a working model, showing how the two proteins might function in plant mitochondria. While HCC1 directly affects COX performance, HCC2 seems to be important for UV-B stress response, possibly by directly or indirectly participating in reactive oxygen (ROS) defense mechanisms.

## Materials and methods

### Plant material and culture conditions

Of the plant lines used in this work, the following were generated previously or obtained from public seed collections, GABI-Kat (German Plant Genomics Research Program—Kölner *Arabidopsis* T-DNA lines; Rosso et al., 2003) and NASC (Nottingham *Arabidopsis* Stock Center; (Scholl et al., 2000)), respectively: *hcc1* (GABI-Kat 923A11; termed *hcc1-2* in Steinebrunner et al., 2011), *hcc2-1* (GABI-Kat 843H01), *hcc2-2* (GABI-Kat 640A10), mt-gk (NASC ID N16263; Nelson et al., 2007), and *HCC1:GUS* line 1 (Steinebrunner et al., 2011). The ecotype Columbia was used as the WT which is also the background of all the mutants used in this study. Sterilized seeds were imbibed overnight at 4°C and then grown on 0.8% (w/v) agar plates (pH 5.7) with 1x Murashige and Skoog basal salts (MS) and 1% (w/v) sucrose. Antibiotics were added when applicable at 25 μg mL^−1^ (kanamycin, hygromycin) and 50 μg mL^−1^ (sulfadiazine). For experiments with adult plants or seed production, seedlings were transferred to soil (Einheitserde, type P, Pätzer, Sinntal-Jossa, Germany) mixed with sand 10:1. The plant growth chamber conditions were 16 h of photosynthetically active radiation (PAR) (100–150 μmol photons m^−2^ s^−1^, determined with a quantum sensor LI-190SA from LI-COR) per day, 35% humidity and 24°C (light)/21°C (dark).

### Bioinformatic analyses

Primers for cloning and genotyping were selected with the help of the SeqViewer tool on the *Arabidopsis* Information Resource database (Rhee et al., 2003). Protein sequences were obtained from the UNIPROT database (www.uniprot.org). Sequence alignment was done with the ClustalO 1.2.0 alignment tool (Sievers et al., 2011; www.ebi.ac.uk/Tools/mhsa/clustalo/). Prediction of transmembrane domains and disulfide bonds was performed with TMPred (Hofmann and Stoffel, 1993; www.ch.embnet.org/software/TMPRED_form.html) and DiANNA (Ferrè and Clote, 2006; clavius.bc.edu/~clotelab/DiANNA/), respectively. Sources for microarray data were the AtGenExpress visualization tool (www.weigelworld.org/expviz/expviz.jsp) and the *Arabidopsis* eFP Browser (Winter et al., 2007).

### Genotypic characterization of *hcc2-1* and *hcc2-2*

The seeds for the lines *hcc2-1* and *hcc2-2* were germinated on MS agar containing sulfadiazine (sul). For genotyping of these and any other mutants used in this work, DNA was isolated as described elsewhere (Steinebrunner et al., 2011). DNA from resistant progeny was subjected to PCR analysis to identify homozygous mutants. The *hcc2-1* and *hcc2-2* alleles were detected with the primer pair 8409/Sco2E1F (330 bp) and 8409/Sco2E5R (570 bp), respectively. The presence of the intact *HCC2* gene was checked with the primer pair Sco2E1F/Sco2E5R. The primer sequences are listed in Supplementary Table 1.

### Generation of GUS reporter lines

The *ABI3:GUS* and *HCC2:GUS* constructs were generated by Gateway cloning (Life Technologies). The *ABI3* (*ABSCISIC ACID INSENSITIVE 3*) promoter region (5 kb) comprised the sequence upstream of the *ABI3* start codon and was amplified with the primer pair ABI3F/ABI3R. The *HCC2* promoter region consisted of 1532 bp upstream of the start codon of *HCC2* and was amplified with the primer pair AtSco2PF/AtSco2PR. All primer sequences contained *attB* recombination sites and are listed in Supplementary Table 1. Each *attB*-flanked PCR product was recombined with the donor vector pDONR221 (Life Technologies) to generate entry clones. The pMDC163 vector (Curtis and Grossniklaus, 2003) which carries the *GUS* (glucuronidase) reporter gene, served as the destination vector for each promoter region present in the entry clone. Plant transformants resulting from *Agrobacterium*-mediated transformation were selected on MS agar plates containing hygromycin (hyg). In addition, the presence of the *ABI3:GUS* construct in the transgenic lines was confirmed by PCR (primers ABI3P-F/pMDC163_GUS-R; Supplementary Table 1).

### GUS activity staining

Plant material was immersed in 90% (v/v) acetone at −20°C for 1 h. After aspirating the acetone, three washes with 50 mM sodium phosphate buffer (pH 7.0) followed. Incubation in 5-bromo-4-chloro-3-indolyl-ß-D-glucuronide (x-gluc) solution [1 mM x-gluc; 50 mM phosphate buffer pH 7.0, 10 mM potassium ferrocyanide, 10 mM potassium ferricyanide, 0.2% (v/v) triton X-100] was done overnight at 37°C. The plant material was transferred to 70% (v/v) ethanol and stored at 4°C.

### Complementation of *hcc1* with *ABI3:HCC1*

For the Gateway cloning (Life Technologies) of the *ABI3:HCC1* construct, PCR products of the *ABI3* promoter region and the *HCC1* sequence which both contained *attB* attachment sites for recombination with suitable donor vectors, were generated. The same *ABI3* promoter region as described for the *ABI3:GUS* construct was used and recombined into the plasmid pDONR221 P1-P5r (Life Technologies). The complete coding sequence of *HCC1* without the stop codon was amplified (primer pair HCC1F/HCC1R) and recombined into the plasmid pDONR221 P5-P2 (Life Technologies). The *HCC1* cDNA U19562 (Yamada et al., 2003) served as the PCR template. The promoterless destination vector pGWB516 (Nakagawa et al., 2007) for the two entry clones *ABI3* promoter and *HCC1* contained four consecutive myc epitope sequences. Therefore, the final *ABI3:HCC1* construct coded for a HCC1 fusion protein that was C-terminally labeled with a 4x myc-tag. Hemizygous *HCC1/hcc1* mutants were transformed with the construct. The progeny were selected on sul and hyg and then genotyped: the *HCC1* transgene was detected with the primers E5F/HCC1E5-6R or E5F/E7R, the genomic *HCC1* allele with the primers E5F/HCC1I6R or E5F/E7R and the *hcc1* allele with the primers E5F/8409. All primer sequences are listed in Supplementary Table 1.

### RT-PCR analysis

Total RNA was extracted from leaves using the peqGOLD plant RNA kit (Peqlab). For reverse transcription, 2 μg of RNA were incubated in a total volume of 9 μL for 5 min at 65°C and then placed on ice for 2 min. The reverse transcriptase M-MuLV (New England Biolabs), oligo(dT) primers, buffer and dNTPs were added in a final volume of 20 μL according to the manufacturer's instructions. The reaction was incubated at 37°C for 60 min and stopped at 70°C for 10 min. The cDNA for the housekeeping gene *UBC21* (ubiquitin-conjugating enzyme 21) was amplified with the primers UBCF/UBCR and for the *HCC2* gene with the primers Sco2E1F/Sco2E5R.

### Complementation of *hcc2-2* with *HCC2:HCC2*

For the complementation of the *hcc2* KO mutant, the entire *HCC2* coding sequence was amplified from *A. thaliana* cDNA (primers Sco2F/Sco2R; Supplementary Table 1) and fused to the Snap-tag (Keppler et al., 2003). The fusion construct was under the control of the identical *HCC2* promoter region used for the *HCC2:GUS* construct. The Gateway technology (Life Technologies) was applied to clone the *HCC2* promoter region and the *HCC2* cDNA into the donor plasmid pDONR P1-P4 and pDONR P4r-P3r, respectively. The cloning of the Snap-tag entry clone is described elsewhere (Steinebrunner et al., 2011). The three entry clones were recombined into the destination vector pGWB516 (Nakagawa et al., 2007). The Snap-tag sequence included a stop codon to avoid fusion with the 4x myc-tag present in pGWB516. The final *HCC2-Snap* sequence coded for a HCC2 protein with a C-terminal 19.6-kDa Snap-tag. Homozygous *hcc2-2* mutants were transformed with agrobacteria containing the *HCC2:HCC2-Snap* construct, hereafter referred to as *HCC2:HCC2*. Transformed progeny were selected on sul and hyg. The presence of the *HCC2* cDNA was confirmed by the detection of a 569-bp amplicon with the primers Sco2E1F/Sco2E5R. The two lines 1–4 and 2–6 used in this work were homozygous for the *HCC2:HCC2* construct and are abbreviated as line 1 and line 2, respectively, hereafter.

### Generation of *HCC2-mRFP* overexpressors

A 1.5-kb *HCC2* gene fragment comprising the sequence from the start codon to 28 nt before the stop codon was amplified with the primers HCC2-BamHIF/HCC2-SalIR, introducing BamHI and SalI restriction sites. The PCR product was cloned into the pENTR 3C dual selection vector (Life Technologies) digested with BamHI and XhoI. Using this clone, the *HCC2* fragment plus the *att* recombination sites from pENTR 3C were amplified by PCR with the oligonucleotides AHL1F/AHL2R. Finally, the PCR product was transferred into the destination vector pGWB554 (Nakagawa et al., 2007) by the Gateway cloning system (Life Technologies). This binary vector contained the constitutive cauliflower mosaic virus *35S* promoter for the expression of the target protein C-terminally fused to monomeric RFP (mRFP). The sequence of the final construct in pGWB554 coded for a HCC2-mRFP fusion protein of 58.1 kDa (30.8 kDa plus the tag of 27.3 kDa). Transformed agrobacteria were used to transform the homozygous kanamycin-resistant *Arabidopsis* mt-gk line (Nelson et al., 2007). This line expresses GFP targeted to mitochondria (mt-GFP). Transformed plants were selected on MS agar containing kanamycin and hyg. The presence of the *HCC2-mRFP* construct was validated by PCR, using genomic DNA and the primer pair HCC2-BamHIF/mCherryR1. In addition, the elevated expression of *HCC2* transcripts in the individual lines was corroborated by qRT-PCR analysis of RNA from roots of 20-day-old seedlings (primer combination HCC2-RT-F/HCC2-RT-R).

### Confocal laser scanning microscopy (CLSM)

For CLSM, the upright Zeiss LSM 780 equipped with water immersion objectives (C-Apochromat 10x/1.20 W Korr M27 or C-Apochromat 63x/1.20 W Korr M27) was used. Roots of 8- to 9-day-old transgenic seedlings co-expressing *HCC2-mRFP* and *mt-GFP* which had been selected on hyg-containing MS agar plates were imaged in water. GFP and mRFP were excited with the 488-nm and 561-nm laser, respectively. Imaging was done in the channel mode choosing 491–552 nm for GFP detection and 587–631 nm for mRFP detection. GFP and mRFP were co-imaged by unidirectional scanning, switching tracks every line. The power of each laser was always kept below the saturation of pixels with the help of the range indicator. The pinhole was set to one airy unit. Bright field-type images were acquired with the transmitted light detector. Crosstalk between channels was ruled out with tissue sections in which individual cells in the same image showed either signals in the GFP or in the mRFP detection channel, but not in both. The images were analyzed with the Zeiss Zen 2010 software.

### Preparation of mitochondria and other subcellular fractions

Mitochondrial crude fractions (MCFs) as well as the fractions SII, PI, and PII were prepared from etioled 10- to 13-day-old seedlings as described previously (Steinebrunner et al., 2011) with slight modifications. The MS medium contained 1% (w/v) sucrose. Baffled flasks were used for better aeration and shaken at 40 rpm. Ethylenediaminetetraacetic acid (EDTA) was added to the grinding buffer to a final concentration of 2 mM. The MCFs were resuspended in 60 μL washing buffer per gram of fresh weight. The *HCC1/hcc1* hemizygotes and the *hcc2-2* KO mutants were grown in the presence of sul. Protein concentrations were determined in duplicates using the bicinchoninic acid protein assay kit from Pierce.

### SDS-PAGE and western blot analysis

Preparation of 10% SDS gels and protein electrophoresis was carried out according to Laemmli (1970). The “PageRuler Plus Prestained Protein Ladder” (Thermo Scientific) was applied as a molecular weight marker. Proteins were transferred onto a polyvinylidene difluoride (PVDF) membrane (Millipore), incubated with primary antibodies, and detected with horseradish peroxidase-conjugated secondary antibodies and the ECL Plus Western blotting detection reagents (GE Healthcare). Primary polyclonal antibodies were diluted in 5% (w/v) nonfat dry milk 1:5000 against mRFP (Rockland), 1:2000 against COX2 (cytochrome *c* oxidase subunit 2; Agrisera), 1:10,000 against RbcL (large subunit of Rubisco; Agrisera) and 1:10,000 against VDAC1 (voltage-dependent anion-selective channel protein; Agrisera). Membranes were stripped between consecutive primary antibody incubations with stripping buffer [62.5 mM Tris pH 6.7, 2% (w/v) SDS, 100 mM β-mercaptoethanol] for 30 min at 55°C.

### Measurement of COX activity

As the purity of the different MCFs can vary, the citrate synthase activity (CSA) was chosen as an mt marker to calibrate the content of mitochondria in each MCF. The CSA was determined spectrophotometrically in a 96-well plate. Each well was successively filled with 130 μL TE-buffer (50 mM Tris/HCl, 2 mM EDTA, pH 8.0), 10 μL 10 mM oxaloacetate, 30 μL 5 mM acetyl-coenzyme A and 10 μL 10 mM 5,5′-dithiobis-2-nitrobenzoic acid. The reaction was started by the addition of 100 μg of MCF per well. Increase in the absorption at 412 nm was followed in a TECAN InfiniteM200 plate reader at 25°C. Measurements were performed in duplicates or triplicates. The slope of the linear phase of the reaction was used to determine the activity. The CSA in the WT MCF was used to recalculate the mt protein amounts in the other MCFs (WT CSA/sample CSA × sample protein amount = calibrated sample protein amount). These adjusted protein concentrations were used for the COX activity measurements and in-gel COX activity stainings.

Measurements of COX activity in isolated mitochondria were performed as described by Sweetlove et al. (2007), using a Clark electrode (STRATHKELVIN Oxygen Meter 782 connected to a Mitocell MT200 chamber). Briefly, 500 μL of mitochondria reaction buffer [0.3 mM sucrose, 10 mM TES/KOH pH 7.5, 10 mM KH_2_PO_4_, 3 mM MgSO_4_, 10 mM NaCl, 0.1% (w/v) bovine serum albumin] were supplemented with 200 μg of MCF followed by addition of 10 μL 0.5 M sodium ascorbate and 25 μL 1 mM reduced cytochrome *c* [from horse heart; prepared without using trichloroacetic acid (TCA); Sigma-Aldrich]. The reaction was started by lysing the mitochondria with 2.5 μL 10% (v/v) Triton X-100. The linear decline of oxygen concentration in the chamber was used to calculate COX activity in comparison with the WT.

### Blue native gel electrophoresis (BN-PAGE) and in-gel COX activity staining

The method of BN-PAGE was performed as described (Schägger and von Jagow, 1991; Schägger, 2001). For the analysis, 150 μg mitochondria (MCF) from etiolated plants were lysed with digitonin (detergent:protein ratio of 4:1), and the lysate was applied to 3–13% BN gradient gels. The protein mixture of the “Gel Filtration Calibration Kit High Molecular Weight” (GE Healthcare) was used as a native molecular weight marker. In-gel COX activity staining was performed modified as described by Thomas et al. (1976). BN gels were equilibrated in COX buffer (75 mg/mL sucrose, 50 mM potassium phosphate buffer, pH 7.4) for 1 h followed by replacement with fresh COX buffer containing 1 mg/mL diaminobenzidine (Fluka) and 1 mg/mL cytochrome *c* (from horse heart; prepared without using TCA; Sigma-Aldrich). The formation of brown precipitates was followed over time and photographically documented.

### Quantitative reverse transcriptase-PCR (qRT-PCR) analysis of *HCC2* expression

Aerial tissue (30 mg each) was frozen in liquid nitrogen at various time points after a 60-min UV-B irradiation. RNA was prepared with TRIzol reagent (Life Technologies) according to the instructions of the manufacturer. First strand cDNA synthesis was performed using oligo dTV_(20)_ primers and M-MuLV (RNase H^−^) reverse transcriptase (New England Biolabs) in a final volume of 20 μL according to the manufacturer's instructions. Quantitative PCR (qPCR) was performed with 2 μL of cDNA synthesis reaction, using primers specific for *HCC2* (HCC2-RT-F/HCC2-RT-R) or *PP2AA3* (protein phosphatase 2A subunit A3) (PP2AA3F/PP2AA3R) (Supplementary Table 1). Both primer pairs spanned two introns each. The qPCR was carried out in a 20 μL final volume, containing 1x of DyNAmo flash SYBR green qPCR kit (Thermo Scientific) and run in triplicates in a Mastercycler ep realplex apparatus (Eppendorf). Relative transcript levels were calculated by the comparative C_T_ method (Livak and Schmittgen, 2001). Expression values were calibrated to transcript levels of the reference gene *PP2AA3* as recommended by Czechowski et al. (2005).

### UV-B treatments

For the *HCC2* promoter activity studies, the *HCC2:GUS* seedlings were selected on hyg-containing MS agar plates. The 7-day-old seedlings were exposed to a 1-h UV-B treatment (Sankyo Denki G15T8E lamps; 1.9 μmol m^−2^s^−1^, determined with a LightScout UV meter from Spectrum Technologies) supplemented with PAR [Osram L15W/840 (daywhite) fluorescent lamps; 28 μmol m^−2^s^−1^]. Then the plates with the seedlings were returned to the plant growth chamber and stained for GUS activity 3 and 24 h later, respectively.

For UV-B stress tests on soil, seedlings were grown without antibiotics on MS agar plates for 7 days and then transferred to soil. For each genotype, 30 plants for the control and 30 plants for the treatment were planted. All plants were cultivated as described under “Plant material and culture conditions,” except that after 10 days on soil the treated plants were irradiated daily for 1 h with UV-B light (1.9 μmol m^−2^s^−1^) supplemented with PAR (28 μmol m^−2^s^−1^). The daily UV-B treatment started 6 h after the beginning of the light period. Pictures were taken on day 1 (right before the first UV-B treatment) and weekly (right after the UV-B exposure) until day 21 (=final UV-B irradiation).

### Gene identifiers

The loci of the genes from this study are provided in parentheses: *ABI3* (At3g24650), *HCC1* (At3g08950), *HCC2* (At4g39740), *PP2AA3* (At1g13320), *UBC21* (At5g25760).

## Results

### *A. thaliana* contains two SCO proteins, but only one of them carries the highly conserved Cu-binding motif

**Figure 1:**
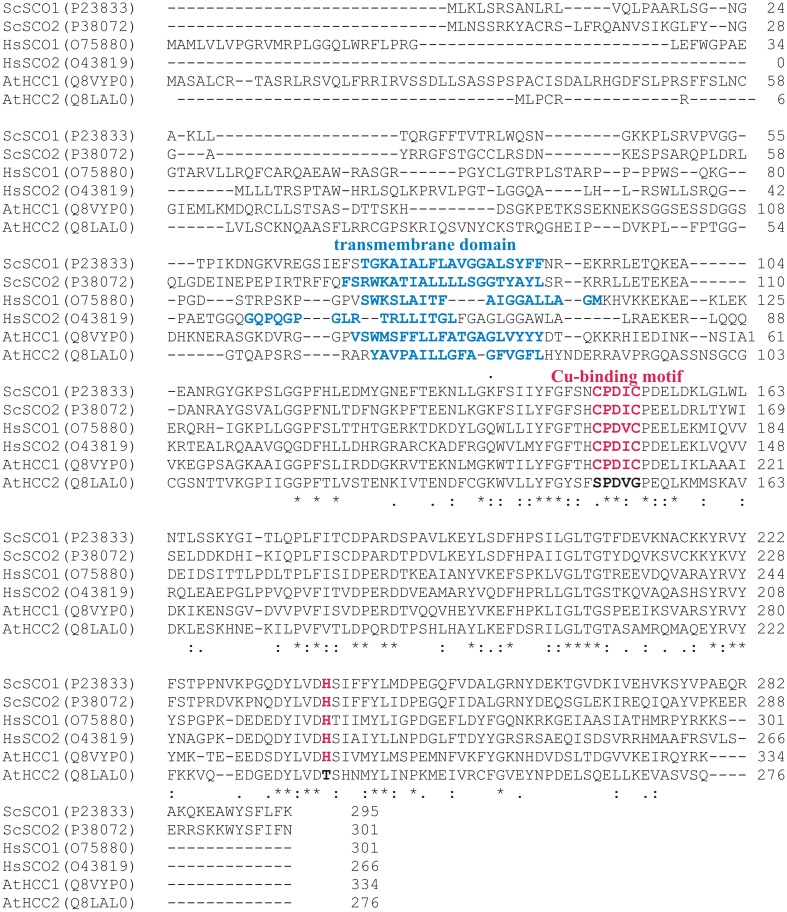
**Protein sequence alignment of SCO proteins from *S. cerevisiae* (ScSCO1 and ScSCO2), human (HsSCO1 and HsSCO2), and *A. thaliana* (AtHCC1 and AtHCC2)**. The alignment was performed with protein sequences from the UNIPROT database (accession codes are given) using the ClustalO 1.2.0 alignment tool (Sievers et al., 2011). The consensus sequence is shown below (“^*^” conserved in all sequences, “.” conserved in some sequences, “:” homologous in all sequences). The predicted transmembrane segments (TMPred; Hofmann and Stoffel, 1993) are shown in blue. The amino acids involved in Cu-binding which includes the CxxxC motif as well as one histidine residue (Rentzsch et al., 1999; Nittis et al., 2001) are marked in red, if present.

The family of SCO-like proteins is highly conserved among prokaryotes and eukaryotes (Arnesano et al., 2005), in which eukaryotes frequently contain at least two of these proteins. Figure 1 shows an alignment of the yeast *S. cerevisiae*, human and *Arabidopsis* SCO proteins. Experimental studies which were mainly performed in yeast unraveled the role of SCO1 in copper transport to the COX complex (Schulze and Rödel, 1988; Rentzsch et al., 1999) and structural analysis revealed the special importance of the CxxxC motif and one histidine residue for copper ligation (Nittis et al., 2001). The members of the protein family in different organisms almost perfectly meet the typical features such as a single transmembrane domain, a high conservation in the C-terminal part, and the Cu-binding motif. However, the latter one is not conserved in HCC2 (Figure 1). Interestingly, one SCO protein containing and one lacking this conserved Cu-binding domain can be found in other higher plants as well (Supplementary Figure 1).

These structural characteristics give hints that HCC2 is rather not involved in copper transport and might have a different function in plants. Therefore, we compared the effects of *HCC1* and *HCC2* loss-of-functions on COX and plant growth performance.

### HCC1 is essential for plant development

**Figure 2:**
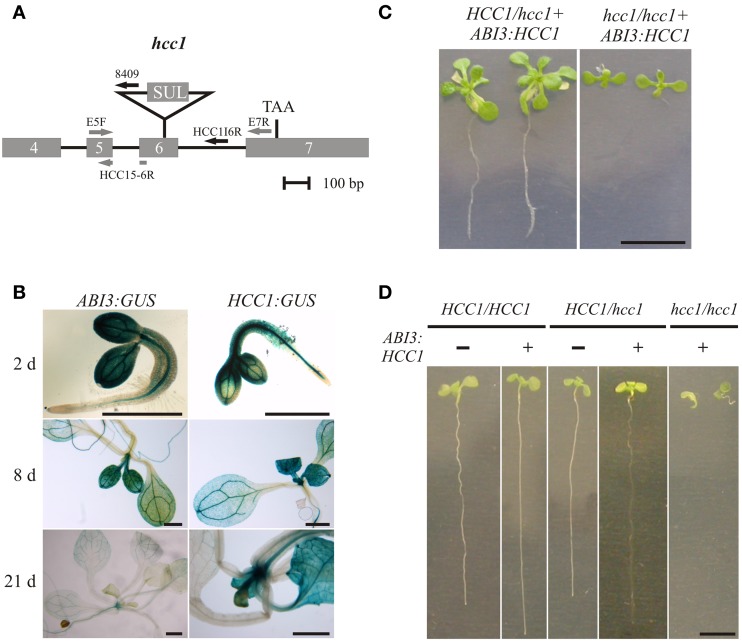
**Embryo-specific complementation of the *hcc1* knockout**. **(A)** A schematic diagram of the *hcc1* allele is shown. The exons 4–7 and introns 4–6 of *HCC1* are denoted by gray boxes and solid lines, respectively. The labeled arrows represent the primers used for genotyping. The gray and black arrow colors denote a binding site in a coding and noncoding region, respectively. Primer HCC1E5-6R spans the exon5-exon6 boundary. SUL, sulfadiazine resistance cassette; TAA, stop codon. **(B)** Seedlings selected on hyg from two *ABI3:GUS* lines (3 and 6) and the *HCC1:GUS* line 1 were stained for GUS activity at the indicated days after germination. The staining pattern was similar in both *ABI3:GUS* lines and representative images of line 3 are shown. The scale bars equal 1 mm each. **(C)** Progeny from a selfing cross of the hemizygous *hcc1* mutant complemented with *ABI3:HCC1* (line “Nils”) were grown on MS agar with hyg and sul for 21 days. The plants were photographed and then used for DNA isolation to determine their genotypes. The scale bar (=1 cm) is valid for both panels. **(D)** Progeny from a selfing cross of the hemizygous *hcc1* mutant complemented *with ABI3:HCC1* (line “Nils”) were grown on an MS agar plate without antibiotics for 13 days. Before each seedling was sacrificed for DNA isolation, the seedlings (sample size = 34) were laid out on agar plates, numbered consecutively and photographed. With the genotyping results at hand, representative images of the WT and the hemizygous *HCC1/hcc1* mutant, both with or without the *ABI3:HCC1* transgene, and of the *ABI3:HCC1*-complemented *hcc1/hcc1* mutant were assembled for the overview. The scale bar equals 5 mm and is valid for all panels.

HCC1 was shown to be essential for embryo development, because T-DNA *hcc1* KO mutants (Figure 2A) died as embryos, mostly at the heart stage (Attallah et al., 2011; Steinebrunner et al., 2011). Previous *HCC1:GUS* analyses revealed *HCC1* promoter activity in seedlings and adult plants, indicating that HCC1 might have a function beyond the embryonic stage (Attallah et al., 2011; Steinebrunner et al., 2011). In order to find out if HCC1 is essential for later stages of plant development as well, a partial complementation strategy was employed. For this, a promoter was needed that would be active during the entire embryo development, but not at any other developmental stage. The *ABI3* promoter was described to meet these criteria (Parcy et al., 1994; Devic et al., 1996). To confirm this, transgenic reporter lines were generated, expressing *GUS* under the control of the *ABI3* promoter. The GUS activity was checked in two *ABI3:GUS* lines at different time points after seedling germination (Figure 2B). In 2-day-old *ABI3:GUS* seedlings, strong GUS staining was visible in cotyledons and in the vasculature of the hypocotyl and root. Eight days after germination of the seeds, the GUS staining was still very strong. After 21 days, however, the GUS activity had considerably faded and only weak staining of the vasculature in older leaves, the hypocotyl and roots remained. The activity of the *HCC1* promoter, on the other hand, was still highly active in the apex and young leaf tissue, substantiating a role of HCC1 in post-embryonic development.

The GUS analyses further demonstrated that the *ABI3* promoter was still active at early stages of seedling growth. The presence of the GUS protein in young seedlings had been reported before by Parcy et al. (1994), although in their analysis, the *GUS* transcript had disappeared by day 4, and the GUS protein, which is more stable, by day 7. For sure, the *ABI3* promoter activity was suppressed at day 21 after seed germination. Therefore, the *ABI3* promoter was selected for the partial complementation approach and fused with the *HCC1* cDNA. Of the two previously characterized T-DNA KO mutants *hcc1-1* (Attallah et al., 2011; Steinebrunner et al., 2011) and *hcc1-2* (Steinebrunner et al., 2011), the T-DNA allele *hcc1-2* (Figure 2A) was chosen for the transformation with *ABI3:HCC1*.

Since the embryo-specific complementation had to be performed in the hemizygous mutant background, nine genotypes were possible as the outcome of the selfing cross (Table 1), assuming that the loci of *HCC1/hcc1* and the transgene were not linked. However, as the *hcc1* KO is embryo lethal, the expected number of viable genotypes in the offspring was only eight.

**Table 1:**
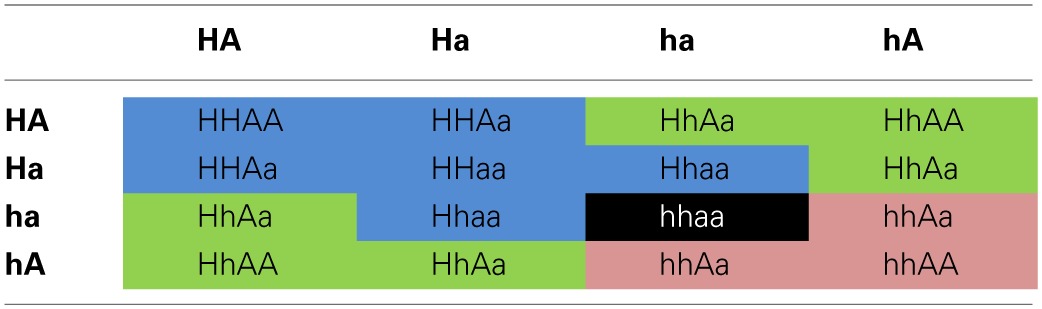
**Punnett square of the selfing cross of the *ABI3:HCC1*-complemented hemizygous *hcc1* mutant**.

*The genotypes of the offspring seedlings sensitive to the antibiotic selection of sul (HHAa, HHAA), hyg (Hhaa), or both (HHaa) are highlighted in blue. The genotypes of the double-resistant WT phenotypes are labeled in green. The genotypes of the double-resistant KO phenotypes are indicated in red. The embryo-lethal genotype is marked in black. H, HCC1; h, hcc1; A, ABI3:HCC1; a, no ABI3:HCC1*.

In order to narrow down the search for the rescued *hcc1* KO mutant, the seeds from the described cross were germinated both on sul and hyg to eliminate all the seedlings that did not carry the *hcc1* mutation and the transgene (Table 1). Thus, 40% of the progeny should get purged. After 21 days of growth, indeed 36% of the 243 offspring plantlets displayed an antibiotic-sensitive phenotype (Table 2). The remaining 155 surviving seedlings could be divided into two phenotypes. One group (45%) had developed several sets of true leaves and a long root, while the other group (19%) had grown only two true leaves and very short roots (Figure 2C; Table 2). The observed percentages of the phenotypes matched the expected percentages (Table 2), indicating that the stunted phenotype represented the complemented *hcc1* KO mutation. As expected, the genotyping of the two phenotypes (Figure 2C) by PCR confirmed that the dwarf plantlets were in fact rescued KO mutants.

**Table 2:**
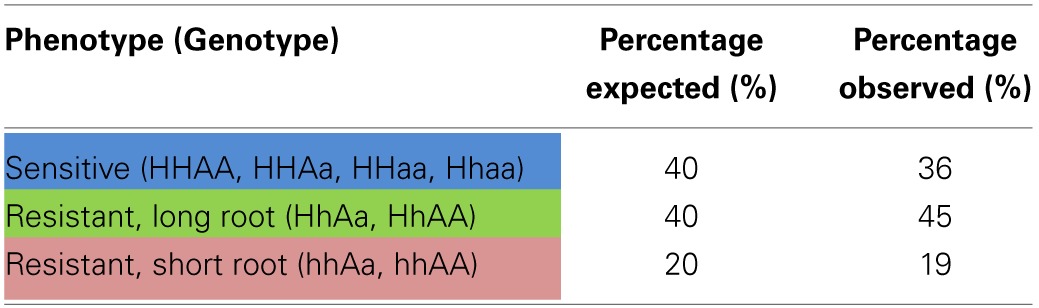
**Segregation analysis of a selfing cross of the *ABI3:HCC1*-complemented hemizygous *hcc1* mutant**.

*The seeds (T2 generation) from a selfing cross of the ABI3:HCC1-complemented hemizygous hcc1 mutant (line “Nils”) were germinated on MS agar containing both sul and hyg. After 21 days the seedlings (n = 243) were sorted by phenotype that was either sensitive or resistant to the antibiotic double selection. The resistant seedlings were subdivided into two groups with long or short roots. The expected percentages for each phenotype group were calculated based on the assumptions that the transgene ABI3:HCC1 and the HCC1/hcc1 loci were not linked and that the transgene was only inserted once into the genome. Because of the embryo-lethality of the allele combination hhaa, the total number of seedling-viable allele combinations was 15 (see Table 1). The deviations of the expected from the observed values were statistically insignificant (p > 0.05; chi-square = 2.5; degrees of freedom = 2). The colors refer to the coding in Table 1. H, HCC1; h, hcc1; A, ABI3:HCC1; a, no ABI3:HCC1*.

In order to rule out that the presence of the transgene exerted a negative effect on plant growth, seeds from an *ABI3:HCC1*-complemented hemizygous selfing cross were germinated on MS agar for 13 days without antibiotics. Thirty-four seedlings were photographed and subsequently genotyped. Now only two different phenotypes appeared: seedlings with long roots or with short roots (Figure 2D). Again, the short roots belonged to the rescued KO mutants. The long roots were grown by all other genotypes, regardless of the presence of the transgene. The phenotype and genotype comparison also showed that the disruption of one *HCC1* gene copy did not impair plant growth.

The *hcc1* KO phenotype became visible as early as day 13 after germination (Figure 2D). The KO mutants did not develop beyond the development of two tiny true leaves and died after transfer to soil (data not shown).

The complementation of the *hcc1* KO mutation with the *ABI3:HCC1* construct demonstrated that *HCC1* is not only essential for embryonic development, but also for plant development in general.

### The disruption of the *HCC2* gene has mild adverse effects on plant growth

The function of HCC1 is obviously crucial for plant development. GUS activity analyses of *HCC2:GUS* transgenic lines (Supplementary Figure 2; Attallah et al., 2011) show staining of mainly the same tissues as observed in *HCC1:GUS* lines (Attallah et al., 2011; Steinebrunner et al., 2011), suggesting similar functions. However, the structural differences (Figure 1) rather pointed to different functions. To resolve this issue, a KO mutant with a T-DNA insertion in intron 2 of the *HCC2* gene (Salk_008313) was studied previously (Attallah et al., 2011). Plants homozygous for the insertion behaved like WT, except for a delayed development of inflorescences (Attallah et al., 2011).

**Figure 3:**
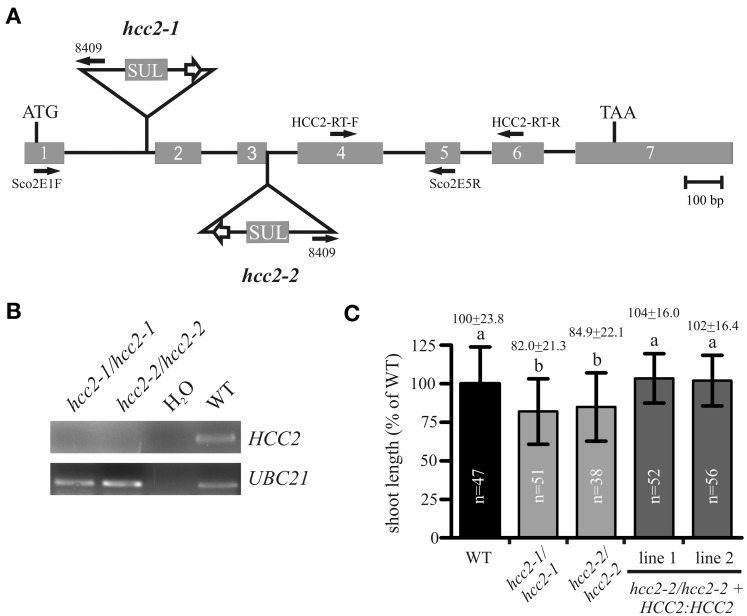
**Characterization of *hcc2* T-DNA mutants**. **(A)** The schematic diagram of the *HCC2* gene shows the insertion sites of T-DNA for the *hcc2-1* and *hcc2-2* mutant, respectively, the orientation and location of primers (solid arrows), the start (ATG) and stop (TAA) codon. Exons (gray boxes) and introns (solid lines) are drawn to scale. The white arrows denote *35S* promoters. SUL, sulfadiazine resistance cassette. **(B)** RT-PCR analysis of *HCC2* expression in homozygous *hcc2* mutants and the WT. Water instead of cDNA was used as a negative control. The amplification of a *UBC21* DNA fragment served as evidence for the presence of cDNA in a reaction. **(C)** The primary stems of 38-day-old plants were measured. The average length (=24 cm) of WT stems was normalized to 100%. Values represent means ± standard deviations (SD). Columns with different letters are significantly different from each other (*p* < 0.01; one-way analysis of variance; Tukey test). The diagram combines the data from two independent experiments. The sample sizes (*n*) are indicated.

In this study, we analyzed two other T-DNA insertion mutants of the *HCC2* gene. One of the two new mutants, *hcc2-1*, bore a T-DNA insertion in intron 1, while the other one, *hcc2-2*, had the T-DNA inserted exactly between exon 3 and intron 3 (Figure 3A). RT-PCR analysis of cDNA from homozygous *hcc2-1* and *hcc2-2* mutants with primers spanning the T-DNA insertions yielded no *HCC2* transcripts, suggesting that both mutants were KOs (Figure 3B). The phenotypes of the homozygous *hcc2* seedlings were indistinguishable from the WT (data not shown). However, after 31 days of growth a slight, but statistically significant (*p* < 0.01) reduction in primary shoot lengths became apparent in comparison with the WT (Figure 3C). In order to confirm that this phenotype was caused by the disruption of the *HCC2* gene, the rescue of the shoot phenotype was pursued by complementing the KO with a functional *HCC2* cDNA fused to the native *HCC2* promoter region. In addition, the *HCC2* cDNA was fused to the Snap-tag (Keppler et al., 2003) to produce a C-terminally tagged HCC2 protein. Two *HCC2*-complemented lines were included in the shoot length analysis and both lines grew primary stems like the WT (Figure 3C), demonstrating that the shoot growth retardation was indeed caused by the *HCC2* loss-of-function.

The *hcc2-2* KO mutant was selected as the background for the rescue experiment for two reasons. First, segregation analysis results available on the website of the GABI-Kat T-DNA mutant collection (Rosso et al., 2003; Kleinboelting et al., 2012) implied that the *hcc2-1* mutant contained at least one more T-DNA insertion locus. Second, qRT-PCR analysis of the *hcc2-1* KO mutant with primers downstream of the known T-DNA insertion revealed about 20-fold higher *HCC2* transcript levels than WT (data not shown; for *HCC2* primer sequences see Attallah et al., 2011). The accumulation of the partial *HCC2* transcripts can be explained by the presence of the suitably oriented *35S* promoter in the T-DNA (Figure 3A). These partial transcripts probably do not give rise to functional HCC2 proteins, because the *hcc2-1* KO mutants are phenotypically identical to the *hcc2-2* KOs (Figure 3C). Nevertheless, all further studies were conducted solely with the *hcc2-2* KO mutant.

The analysis of the two *hcc2* KO mutants corroborated that the importance of HCC2 for plant development, unlike the function of HCC1, is limited under normal growth conditions.

### HCC2 is localized in mitochondria

Several programs predict the localization of both plant SCO proteins in mitochondria (SUBA database; Heazlewood et al., 2007). Different experimental approaches confirmed the mt localization of HCC1. HCC1 was detected in mt fractions by mass spectrometry (Dunkley et al., 2006) and by Western blot analyses, using a Snap-tagged version of HCC1 (Steinebrunner et al., 2011). However, there were no experimental data available for HCC2.

Initially, the same Snap-tagging approach was attempted for HCC2 to prove its presence in mitochondria. However, it was impossible to detect the biotinylated HCC2-Snap protein in mitochondria (data not shown). One explanation for the detection failure could be that HCC2 is less abundant than HCC1. In fact, studies in the yeast *S. cerevisiae* revealed that there are 1.7 times more Sco1p than Sco2p molecules per cell (Ghaemmaghami et al., 2003).

As an alternative strategy to investigate the predicted localization, HCC2 was C-terminally fused to mRFP and expressed under the control of the *35S* promoter. The construct was transformed into a transgenic line which expressed GFP targeted to mitochondria (mt-GFP) (Nelson et al., 2007).

**Figure 4:**
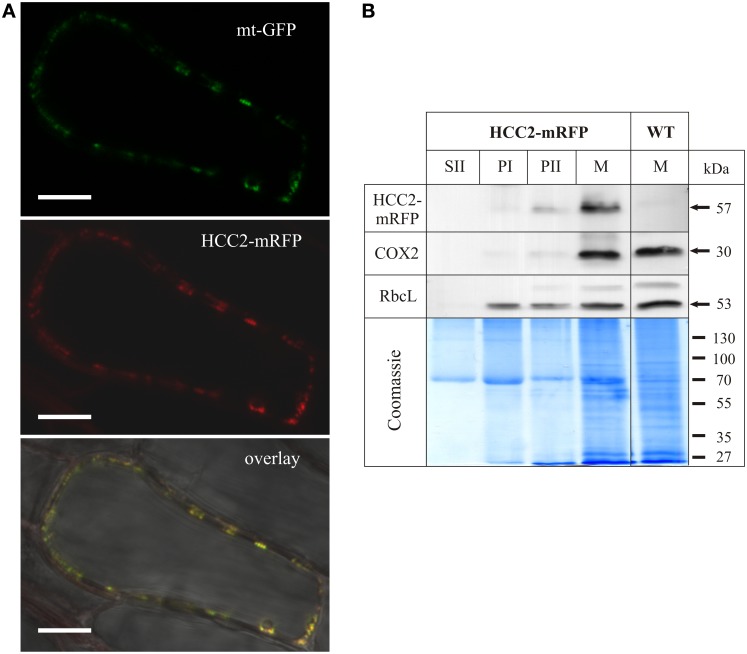
**Subcellular localization analysis of HCC2-mRFP. (A)** Five different transgenic lines co-expressing *HCC2-mRFP* and GFP targeted to mitochondria (mt-GFP) were imaged by laser scanning microscopy, and co-localization of HCC2-mRFP and mt-GFP was found in each line. A representative cell (line 3) is shown, in which co-localization of the green mt-GFP (top panel) and the red HCC2-mRFP signals (middle panel) is depicted as yellow signals after merging the two images (overlay). The overlay also includes a bright-field image of the cell. The image was acquired with a resolution of 1024 × 1024 pixels and a pixel dwell of 3.15 μs. Scale bars correspond to 10 μm each. **(B)** Mitochondria (M), supernatant (SII) and pellet fractions (PI, PII) were prepared as described in Material and Methods from light-grown WT seedlings or *HCC2-mRFP* overexpressors (line 5) selected on hyg. The indicated fractions (50 μg each) obtained during the preparations were subjected to 10% SDS-PAGE. Western blot analyses of the same membrane were performed with antibodies directed against mRFP, cytochrome *c* oxidase subunit 2 (COX2) and the large subunit of Rubisco (RbcL). The total protein in the gel was visualized after the transfer by colloidal Coomassie staining.

Roots of various transgenic lines co-expressing *HCC2-mRFP* and *mt-GFP* were imaged by confocal laser scanning microscopy (CSLM). Numerous dot-like and often fast-moving structures were visible in the cytosol, using the GFP detection channel (Figure 4A, top panel). The diameter of these GFP signals was approximately 0.5 μm, matching the size of *Arabidopsis* mitochondria (Nelson et al., 2007). When detecting the mRFP in the same cells, the fluorescence signal pattern was very similar (Figure 4A, middle panel), suggesting co-localization. Indeed, the GFP and mRFP signals nicely overlapped (Figure 4A, bottom panel), suggesting that HCC2 is localized in mitochondria. No mRFP signal was detectable in any other compartments inside or outside of cells.

In order to confirm the localization results, Western blot analyses of different fractions obtained during the preparation of mitochondria (M) from *HCC2-mRFP* expressing lines and the WT control were performed (Figure 4B). Identity of the different fractions was confirmed by the detection of COX2 and RbcL as mt and chloroplastic marker proteins, respectively. The COX2 and RbcL proteins were absent in the supernatant fraction II which is expected to contain soluble proteins and to be organelle-free. The chloroplast marker RbcL was present in all pellet fractions (PI, PII, M). The mt marker COX2, on the other hand, was faintly detectable in PII, but strongly enriched in the mt fraction (M).

Immunological detection using an mRFP antibody yielded a weak signal in the PII fraction and a strong signal in the mt fraction of the *HCC2-mRFP* overexpressing line, but not of the WT which served as a control for the specificity of the antibody. The molecular weight (~57 kDa) of the signal corresponded to the theoretical size of HCC2 (31 kDa minus 1 kDa signal peptide) fused to mRFP (27 kDa). The molecular weight of the signal also demonstrated that the HCC2-mRFP protein exists *in planta* and that the fluorescence detected by CLSM was not originating from free mRFP.

The signal pattern for COX2 and HCC2-mRFP matched, indicating the mt localization of HCC2-mRFP. The missing signal for HCC2-mRFP in the fraction PI, which showed a strong signal for RbcL, argues against an additional localization of this protein in chloroplasts.

Our data obtained by two independent methods give experimental evidence for the localization of HCC2 in plant mitochondria.

### COX activity is severely affected in *HCC1/hcc1* hemizygotes, but not in *hcc2* KO mutants

SCO proteins are hypothesized to function in the copper transport and/or assembly of the COX complex in eukaryotes. Indeed, previous results using diaminobenzidine staining showed no COX activity in *hcc1-1*/*hcc1-1* embryos (Attallah et al., 2011). However, since these embryos do not develop into adult plants, it is impossible to further investigate the COX complex assembly in the KO background. Therefore, hemizygous *HCC1*/*hcc1* plants were chosen to see if the deletion of one *HCC1* gene copy was sufficient to cause COX alterations. In order to analyze a possible influence of HCC2 on COX function, the *hcc2-2* KO was included in the study. From these mutants as well as the WT, mt fractions were prepared and the amount of mitochondria in these fractions was equilibrated by measuring CSA (data not shown).

In *S. cerevisiae*, a lack of Sco1p results in not only missing COX activity, but also destabilization of the complex and degradation of the COX subunits (Paret et al., 2000). Therefore, the protein level of COX2 was previously investigated in the *hcc2* Salk KO mutant and found to be unaffected (Attallah et al., 2011). The Western blot analysis was also done with the *hcc2-2* KO to confirm that COX2 levels were independent of the presence of functional HCC2 proteins. Clearly, the amount of COX2 was not reduced in the *hcc2-2* KO compared with the WT (Supplementary Figure 3), providing a first hint that HCC2 is not directly involved in COX assembly.

**Figure 5:**
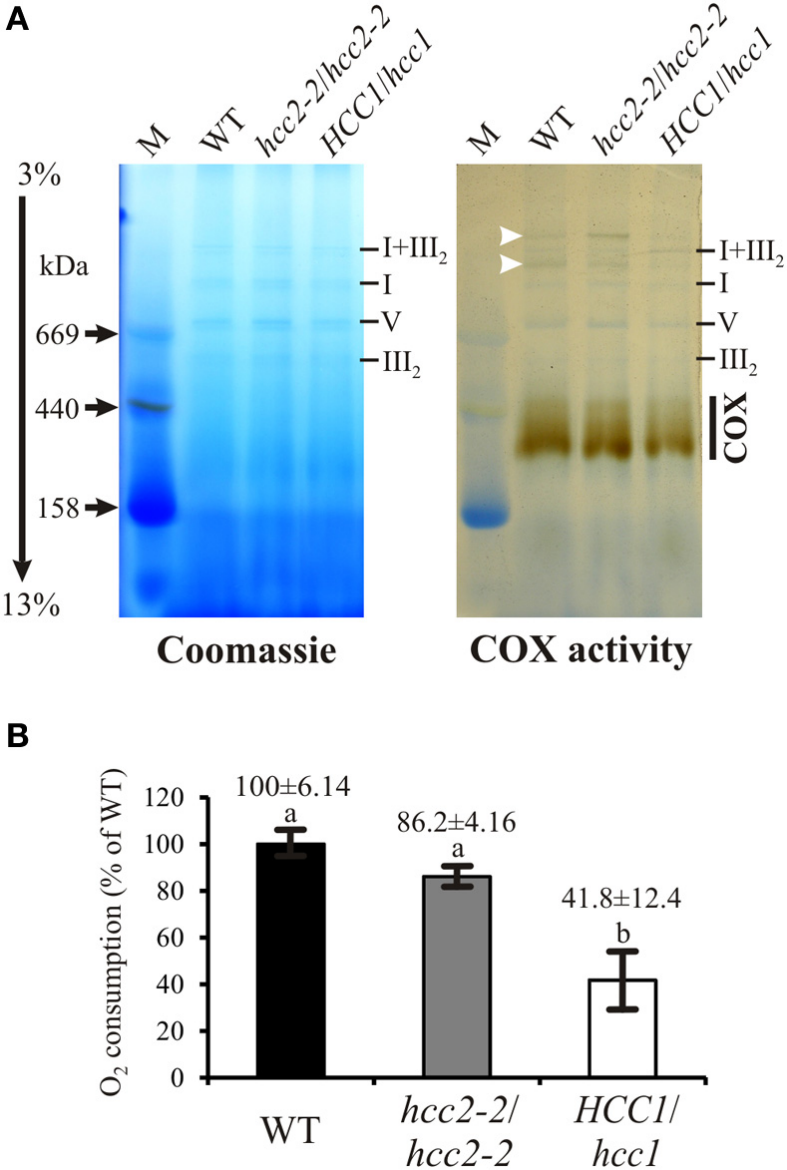
**COX activity analysis in the WT, *hcc2* KO and hemizygous *HCC1/hcc1* mutants**. **(A)** Mitochondria (150 μg each) from WT and mutant etiolated seedlings were solubilized using digitonin. The protein complexes were separated in a 3–13% gradient gel by BN-PAGE (left panel). COX complex activity was visualized in the same gel (right panel) as described in Material and Methods. The positions of abundant respiratory chain complexes (III_2_, V, I, and I+III_2_) were determined by comparison to the BN electrophoretic mobility analysis of Klodmann et al. (2011). The white arrowheads mark putative supercomplexes explained in the Results section. M, molecular weight marker. **(B)** COX activity in mitochondria was determined by measuring oxygen consumption with a Clark electrode after addition of reduced cytochrome *c*. Mean values derive from triplicate measurements of two independent mt preparations. Error bars indicate standard deviations. The mean WT activity (=O_2_ consumption of 24 μmol L^−1^ min^−1^) was set to 100%. Different letters indicate means that are significantly different from each other (*p* < 0.01; one-way analysis of variance; Tukey test).

For a more direct analysis of a link between the HCC proteins and COX, its activity was measured by two different approaches. First, blue native gel electrophoresis (BN-PAGE) allowed us to study COX activity in conjunction with the molecular organization of the complex (Figure 5A). The in-gel staining revealed COX activity in a broad molecular range of about 300–450 kDa (Figure 5A, COX), in which the strongest band was visible in the lower range of the stained area. The activity in the *hcc2-2* KO mutant was very similar to that in the WT, whereas the staining intensity seemed to be weaker in the hemizygous *HCC1*/*hcc1* plants. The molecular organization of the complex in this molecular weight range did not seem to be different among the three genotypes.

Interestingly, also two light-brownish bands in the range above 1 MDa could be detected in the WT and the *hcc2-2* KO, but not in the *HCC1*/*hcc1* mutant (Figure 5A; white arrowheads). These bands might represent COX associations with other complexes of the respiratory chain (so-called supercomplexes), which were described in several organisms (Schägger, 2002) including the plants potato, spinach and asparagus (reviewed in Dudkina et al., 2008). In *Arabidopsis*, only respiratory chain supercomplexes consisting of complex I and III have been described so far (Eubel et al., 2003; Klodmann et al., 2011). However, these previous studies differed strongly in their experimental design from our analysis, since they used suspension cell culture as starting material and harsher lysis conditions of the mitochondria. This might also explain why we detected activity deriving from monomeric—and possibly dimeric—COX at a slightly higher molecular weight range (up to 450 kDa) than in these publications (at 200–230 kDa).

Whether the higher molecular weight complexes we detected indeed contain COX and under which physiological conditions these supercomplexes are present, remains to be elucidated.

Although the BN results gave hints that the COX activity was reduced in the *HCC1*/*hcc1* mutant, but not in the *hcc2-2* KO, the staining was not suitable for quantification of the enzyme activity. Therefore, COX activity measurements were performed in isolated mitochondria, using a Clark electrode as the second approach (Figure 5B). Our results showed that the activity was only minimally reduced in the *hcc2-2* KO (~14%) compared with the WT, and the difference was not statistically significant. In the hemizygous *HCC1*/*hcc1* mutant, however, it was suppressed by almost 60% relative to the WT.

These data validate the proposition that HCC1 is crucial for COX function, since the deletion of one *HCC1* gene copy already leads to a severe drop in COX activity. Furthermore, the data suggest that the homozygous knockout of *HCC2* does not affect COX activity and strengthen the hypothesis that this protein is involved in a different cellular pathway in *Arabidopsis*.

### *HCC2* KO mutants are more sensitive to UV-B stress

**Figure 6:**
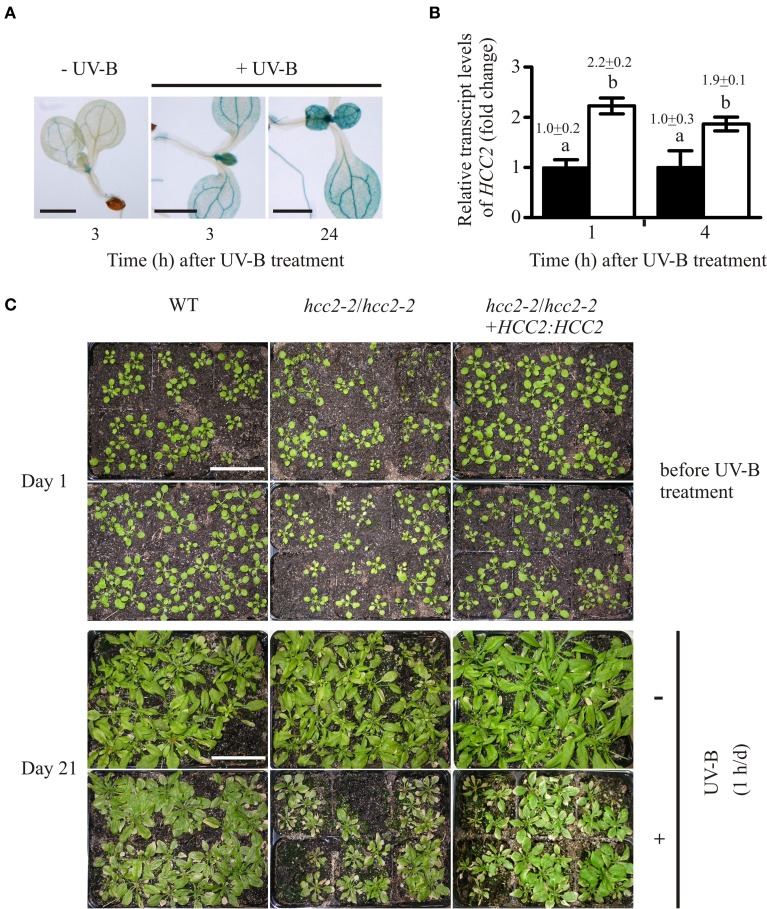
**Response of *hcc2* mutants and WT to UV-B treatment**. **(A)** *HCC2:GUS* seedlings were stained for GUS activity 3 h or 24 h after a 1-h treatment with UV-B light. Untreated seedlings were stained in parallel to the 3-h time point. Two GUS lines (lines 3 and 7) were included in the experiment, and three seedlings were photographed per time point. Here seedlings of the line 3 are shown. The experiment was repeated once providing the same staining pattern. The scale bars equal 1 mm each. **(B)** The relative amounts of *HCC2* transcripts were analyzed in 14-day-old WT seedlings by qRT-PCR which were either treated with UV-B for 1 h (white bars) or left untreated (black bars). RNA was isolated from leaves harvested at the indicated time points. The amounts of *HCC2* transcripts at time point 1 h of the untreated control were set to 1. *PP2AA3* transcript levels were used as a reference for normalization. Error bars represent standard deviations calculated from the normalized *HCC2* expression values of two independent RNA preparations of the same UV-B assay. Different letters denote values which are statistically significantly different from each other (*p* < 0.05; one-way analysis of variance; Tukey test). *HCC2* levels shown in this graph are representative of two independent UV-B experiments. **(C)** Day 1 marks the beginning of a UV-B stress test in which 17-day-old WT plants, *hcc2-2* KOs and complemented *hcc2-2* KOs (line 2) either served as untreated controls (top row) or were irradiated daily with UV-B light for 1 h (bottom row). The phenotypes of the same plants are shown after they had grown for 21 days in the absence of any UV-B light (-UV-B) or exposed daily to UV-B (+UV-B). Any plants which died during the UV-B stress test were removed. At day 21, the shoots were cut for unobstructed view of the rosettes. The scale bars equal 5 cm each and are valid for all panels. This UV-B assay was conducted three times showing the same phenotypic responses.

Public databases of microarray data indicated that *HCC2* is induced by UV-B light. *HCC2* transcript levels were approximately two times higher 1 h and 3 h after UV-B irradiation than at time point zero (AtGenExpress visualization tool; *Arabidopsis* eFP Browser). For confirmation, *HCC2:GUS* seedlings were irradiated with UV-B light for 1 h and stained for GUS activity at subsequent time points. At 3 h as well as 24 h after the UV-B treatment, the GUS staining was noticeably stronger compared with the untreated control (Figure 6A), showing that UV-B light stimulated *HCC2* promoter activity.

Additionally, we wanted to confirm the increase of *HCC2* transcript amounts by UV-B, especially under our treatment conditions. The plants were started off on MS agar for 7 days and let continue to grow on soil for 10 days. For the *HCC2* transcript analysis, RNA samples were taken shortly after the UV-B treatment to minimize possible stimulation of *HCC2* expression by secondary effects such as tissue damage. *HCC2* transcript amounts increased about two-fold 1 h after the UV-B exposure ended and remained at this level 4 h later (Figure 6B). This increase lies within the same range observed after a 15-min UV-B treatment under similar conditions (AtGenExpress visualization tool; *Arabidopsis* eFP Browser; Kilian et al., 2007).

In order to see if the UV-B-induced activation of *HCC2* transcription had a beneficial effect, the performance of the *hcc2-2* KO and WT was compared during a 3-week-long UV-B stress test (see Material and Methods). The 17-day-old plants were treated daily with UV-B light. Four daily UV-B exposure times (15 min, 30 min, 45 min, 60 min) were tested. Of the different exposure times, 15 min turned out to cause no visible damage (data not shown). The longer exposures led to yellowing and stunting of rosette leaves and sometimes even plant death. Sixty minutes allowed the best distinction of phenotypic differences among the various genotypes compared.

All genotypes were negatively affected by the treatment as the comparison with the untreated plants demonstrated, but the WT was more tolerant to the stress than the *hcc2-2* KO (Figure 6C). Complementing the *hcc2-2* KO with a functional *HCC2* cDNA remedied the heightened UV-B sensitivity (Figure 6C).

Our data show that *HCC2* is upregulated by UV-B exposure and that a deletion of *HCC2* increases the UV-B-sensitivity of the plants.

## Discussion

The genomes of seed plants encode two proteins with sequence similarity to SCO proteins from other organisms. Even though it has been postulated that SCO proteins may have additional functions, little information is available about the involvement of SCO proteins in processes unrelated to COX assembly. In the present work, we investigated the functions of HCC1 and HCC2, the two SCO proteins from *A. thaliana*, through the analysis of a variety of mutants.

### HCC1 is essential for COX activity

For HCC1, the presence of conserved amino acids (aa) which were demonstrated in *S. cerevisiae* to be required for copper binding (Rentzsch et al., 1999; Nittis et al., 2001) implied that HCC1 can bind Cu. qRT-PCR analyses showed that *HCC1* transcript levels increased after copper treatment, analogous to the rise of other copper chaperone transcript amounts by copper (Del Pozo et al., 2010). Genetic evidence for a role in Cu binding and delivery was provided by complementation studies of the yeast deletion mutant *Δsco1* with a gene encoding a chimeric ScSco1p-HCC1 fusion protein. The rescue of the mutant was enhanced by Cu supplementation (Steinebrunner et al., 2011). Since the yeast *Δsco1* mutant was respiratory-deficient (Schulze and Rödel, 1988), the copper chaperone function of HCC1 seemed to be essential for respiration. However, only indirect experimental evidence existed for this role in *Arabidopsis* (Attallah et al., 2011). This work unequivocally shows that the *HCC1* loss-of-function influences COX activity, as the disruption of one WT *HCC1* allele was enough to lower the COX activity to more than half of the WT level. It would be interesting to see if the HCC1 protein levels and COX activity directly correlate or if even a small reduction in HCC1 amounts is enough to compromise COX function.

A reduction of COX activity (~20%) was observed in a *S. cerevisiae* mutant with a point mutation in *SCO1*, but despite the suppressed respiration capacity the yeast cells continued to grow on nonfermentable carbon sources like the WT (Lode, 2001). Similarly, the strong effect on COX activity did not seem to affect plant growth. Plants with only one intact *HCC1* copy looked phenotypically indistinguishable from the WT (Figure 2D). In agreement with this, COX-deprived mutants of the green alga *Chlamydomonas reinhardtii* continued to grow like the WT when kept in the light (Colin et al., 1995; Remacle et al., 2010). Apparently, the reduced production of ATP is compensated by photosynthesis, or other metabolic pathways such as glycolysis may contribute some ATP. The latter process might be enhanced by the presence of sucrose in our growth media. In future studies it might therefore be interesting to compare the adenylate status of the different mutant lines under various growth conditions.

However, COX activity is linked to many important processes other than the provision of ATP. For example, it maintains the mt membrane potential necessary for the import of many mt proteins from the cytosol (reviewed in Chacinska et al., 2009). In addition, the synthesis of ascorbate is dependent on COX activity. The oxidation of L-galactono-γ-lactone to ascorbate by L-galactono-γ-lactone dehydrogenase uses cytochrome *c* as an electron acceptor. Therefore, a continuous electron flow from cytochrome *c* to complex IV is prerequisite for ascorbate biosynthesis (Bartoli et al., 2000). Ascorbate affects growth, because low levels correlate with low cell division rates (Kerk and Feldman, 1995). In addition, ascorbate is a co-factor for prolyl hydroxylases (Smirnoff, 2000) which produce hydroxyproline-rich glycoproteins relevant for cell wall structure (Showalter, 2001). This function might be the reason why *HCC1* promoter activity is not only high in metabolically active cell types, but also in trichome support cells (Steinebrunner et al., 2011) which need mechanical reinforcements. This function in ascorbate synthesis could also explain why *Arabidopsis* mutants without functional cytochrome *c* (Welchen et al., 2012) and *HCC1* (Attallah et al., 2011; Steinebrunner et al., 2011) die as embryos. For the *hcc1* KO embryos, the predominant time point of their developmental arrest was pinned to the heart stage (Steinebrunner et al., 2011). At this stage, the embryos start growing anisodiametrically, forming two symmetric protrusions as they transit into the torpedo stage. The cell wall plays a pivotal role for these local cell divisions and expansions as proposed for the cell wall mutant *cyt1*. The affected *CYT1* gene codes for the precursor of cell wall polysaccharides and ascorbate (reviewed in Smirnoff, 2000) and the knockout mutant also stops growing as a heart-shaped embryo (Lukowitz et al., 2001).

### Possible redox roles of HCC2

The homolog HCC2 does not contain the conserved Cu-binding motif, contradicting a function as a copper chaperone. However, its promoter activity overlapped with the *HCC1* promoter activity (Attallah et al., 2011; Steinebrunner et al., 2011; Supplementary Figure 2), hinting at similar functions. However, the determined WT levels of COX activity in the *hcc2-2* KO mutant provided compelling evidence that HCC2 is not required for COX function.

No effect on respiration efficiency was also documented for the deletion of *SCO2* in yeast (Glerum et al., 1996). Instead, an indirect involvement of Sco2p in COX assembly was postulated. The two cysteines of the Cu-binding motif in the thioredoxin domain of Sco2p could possibly maintain the proper redox state of Sco1p, allowing Sco1p to bind and release Cu (Banci et al., 2007). In human cells it could indeed be shown that SCO2 acts as thiol-disulfide oxidoreductase for SCO1 (Leary et al., 2009).

However, HCC2 strikingly lacks these cysteines as well as the histidine residue contributing to Cu binding. The general occurrence of a SCO homolog that bears no conserved amino acids relevant to Cu binding seems to be a common feature in plants (Supplementary Figure 1), arguing against its function in metal transfer. But what could be such a plant-specific function?

The results presented here suggest a role in the UV-B stress response, however, it is not clear if HCC2 specifically responds to UV-B or if it fulfills a general function as an antioxidant through its thioredoxin-like fold. In favor of a UV-B-specific function is the finding that (i) *HCC2* is upregulated by UV-B treatment and that (ii) HCC2 is not upregulated by UV-A or by other factors causing oxidative stress such as paraquat (Kilian et al., 2007). These data should be confirmed and complemented with phenotypic studies in the future. Preliminary studies indicate that *hcc2* KO plants contain higher lipid peroxidation levels under basal growth conditions, suggesting that HCC2 could serve as a regulator of ROS levels.

The homologous yeast proteins Sco1p and Sco2p were both shown to reside in the inner mt membrane and to protrude their catalytically active C-terminal domain into the intermembrane space (IMS) (Krummeck, 1992; Lode et al., 2002). The data of our work do not allow conclusions about the submitochondrial localization of HCC2, but nicely prove its presence in mitochondria.

Nevertheless, the analysis tool TMPred predicts for HCC2 one transmembrane helix from aa 66 to 82 with an inside-outside orientation, arguing for a localization of the active domain of HCC2 in the mt IMS space. This subcompartment represents a suitable site for a protein with an oxidoreductive function, because the IMS contains the highest abundance of cysteine-rich proteins in the cell (Herrmann and Funes, 2005).

### Model for HCC1 and HCC2 function

**Figure 7:**
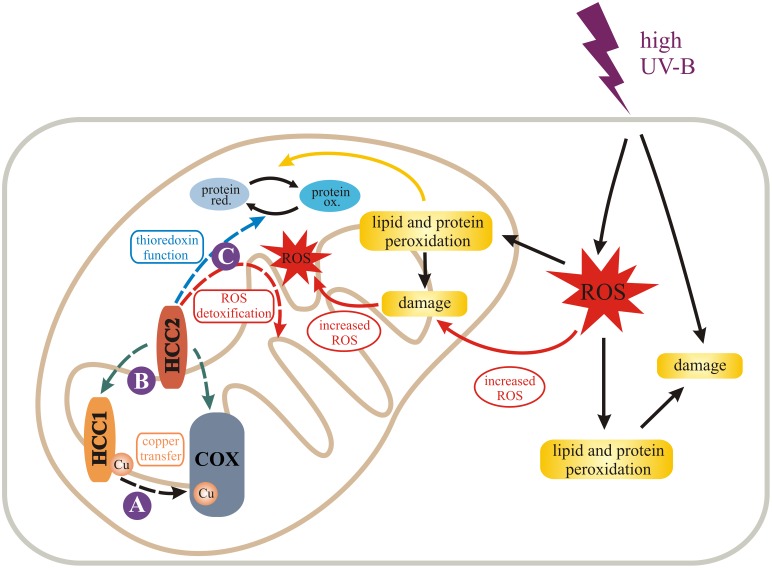
**Model for the role of HCC1 and HCC2 in plant mitochondria**. We propose different functions (dashed arrows) for the two SCO proteins in mitochondria of *A. thaliana* indicated by solid purple circles. HCC1 is an essential plant protein and is most likely involved in copper transport to COX (complex IV) of the respiratory chain (A). HCC2 might have rather a slight influence on COX activity by either directly or indirectly supporting HCC1 function (B). In fact it is more likely that HCC2 functions as a thioredoxin, converting oxidized (ox.) proteins back to their reduced (red.) forms, or detoxifies reactive oxygen species (ROS) in mitochondria (C). This function is of special importance under UV-B stress which leads to increased ROS damage in cells and organelles, e.g., by lipid and protein peroxidation. The figure was partially adapted from Nawkar et al. (2013).

Incorporating our experimental data, we propose the following model (Figure 7). HCC1 is essential for plant life, because it ensures COX function, most conceivably through delivery of the co-factor Cu to complex IV. HCC2, on the contrary, is not an essential plant protein, but important nonetheless. As a putative oxidoreductase, HCC2 could maintain the proper redox state of redox-sensitive proteins, such as HCC1, although COX activity was not significantly suppressed in the *hcc2-2* KO. However, the HCC2 activity is possibly compensated in the mutant by an mt oxidoreductase of redundant function, or HCC2 may only be important under certain conditions, such as UV-B stress.

High UV-B fluence rates—as used in our experiments—trigger the production of ROS (reviewed in Mackerness et al., 2001 and Hideg et al., 2013) which cause oxidative damage of cell components. The damage leads to the release of more ROS, amplifying the original ROS levels. HCC2 could protect against ROS damage by exerting the oxidoreductive function of its thioredoxin domain, protecting the plant indirectly against UV-B. However, as a caveat for this hypothesis it must be considered that despite the fact that HCC2 contains a thioredoxin fold, the typical thioredoxin motif CxxC directly involved in catalysis (reviewed in Meyer et al., 2012) is missing. Nevertheless, HCC2 is a very cysteine-rich protein with five cysteines remaining in the protein after the putative signal peptide (aa 1–29) is cleaved off. In addition, there is a CGC-motif (aa 102–104) which is predicted to form a disulfide bond with C_253_ by the DiANNA software. Additionally, any of the other cysteines could exert a redox-active function. Alternatively, HCC2 may not act directly as an oxidoreductase, but may modify the activity of other redox proteins present in the mitochondrial IMS.

In conclusion, plants have evolved two different SCO proteins with specific functions in COX assembly and stress responses. Even though our results indicate that HCC1 and HCC2 have completely separate roles, the presence of both proteins in mitochondria and the conservation in structure does not rule out that they exert their functions through partially overlapping pathways. The results highlight the role of mitochondria in many physiological responses and indicate that plants have adapted preexisting proteins to serve additional functions related to their specific needs.

## Author contributions

Iris Steinebrunner conceived of the study, characterized mutant lines (*HCC2:HCC2, hcc2-1, hcc2-2, hcc1* lines complemented with *ABI3:HCC1* or *ABI3:GUS*), did the confocal microscopy and segregation analyses and drafted the manuscript. Uta Gey conducted the COX activity experiments, the BN-PAGE and Western blot analyses, performed the sequence alignments and participated in the writing of the manuscript. Manuela Andres did the UV-B experiments and analyzed the *GUS* expression controlled by the *HCC2* promoter in response to UV-B light and by the *ABI3* promoter. Lucila Garcia generated the *35S:HCC2-mRFP* lines and did the qRT-PCR analyses. Daniel H. Gonzalez advised the study and participated in the writing. All authors read and approved the final manuscript.

### Conflict of interest statement

The authors declare that the research was conducted in the absence of any commercial or financial relationships that could be construed as a potential conflict of interest.

## Supplementary material

Supplementary Table 1 **Primer details**. ^*^The attachment site sequences necessary for recombination were omitted. Only the gene-specific sequences are listed.

Supplementary Figure 1 **Protein sequence alignment of SCO (HCC) proteins from plants: *Arabidopsis thaliana* (At), *Oryza sativa* subsp. *japonica* (Os), *Brassica rapa* subsp. *pekinensis* (Br), *Glycine max* (Gm), and *Solanum lycopersicum* (Sl)**. Protein sequences were retrieved from the UNIPROT database (UNIPROT numbers are given). The sequence alignment was performed using the ClustalO 1.2.0 alignment tool (Sievers et al., 2011). The consensus is quoted below the sequences (“^*^” conserved in all sequences, “****.” partially conserved, “**:**” homology in all sequences). The amino acids involved in Cu-binding which includes the CxxxC motif as well as one histidine residue (Rentzsch et al., 1999; Nittis et al., 2001) are marked in red, if present. Proteins containing these conserved residues are titled “HCC1,” whereas those missing the motif are named “HCC2.” If more than one sequence in the UNIPROT database met this criterion, they were distinguished by different letters (a, b, c).

Supplementary Figure 2 ***HCC2:GUS* studies of different developmental stages**. Various tissues from *HCC2:GUS* transgenic lines were stained for GUS activity: Ovules **(A,B)**, embryo **(C)**, cotyledon **(D,E)**, roots **(F,G)**, leaves **(H,I)**, flower buds **(J)**, flower **(K)** and silique **(L)**. The tissues stemmed from 7-day-old seedlings **(D–G)** and 4-week-old plants **(A–C, H–L)**, respectively. Exemplary guard cells are indicated by arrows in **(E)**. Staining of guard and trichome support cells was only observed in younger leaves. ch, chalaza; f, funiculus; r, root; h, hypocotyl; tsc, trichome support cells; c, connective; az, abscission zone.

Supplementary Figure 3 **Analysis of steady-state COX2 levels in the WT and *hcc2-2* KO mutants**. MCF (50 μg each, normalized to WT CSA levels) prepared from liquid cultures of etiolated seedlings (WT and *hcc2-2*/*hcc2-2* mutants) were separated in a 15% SDS gel for Western blot analysis. Immunological detection of COX2 and VDAC1 was performed successively on the same membrane. VDAC1 was detected as the loading control for equal amounts of mt protein. The Western blot analysis was performed three times with two independent sets of protein extracts, producing the same results.
