# Supplementary material for: Divergent functions of the Arabidopsis mitochondrial SCO proteins: HCC1 is essential for COX activity while HCC2 is involved in the UV-B stress response
Source: Front Plant Sci. 2014 Mar 25;5:87. doi: 10.3389/fpls.2014.00087 (PMC3971200; doi:10.3389/fpls.2014.00087)
Supplement: Supplementary Table 1 — Primer details. *The attachment site sequences necessary for recombination were omitted. Only the gene-specific sequences are listed. [file DataSheet1.PDF]

Supplementary Material

Supplementary Table 1: Primer details

Primer name	Sequence (5'→3')	Amplicon size
ABI3F	CCGGAGTTAGAGACCTGA*	5 kb
ABI3R	CGTTGAAGTGGAAATGAAAC*	
AtSco2PF	CTCAACCTTGTGCGCATC*	1590 bp
AtSco2PR	TGAATTTGGTTCTTCTTCTC*	
HCC1F	ATGGCGTCTGCTCTATGTAGA*	1002 bp (cDNA)
HCC1R	ACTTCCGGTACTGACGGATC*	
ABI3P-F	GGTAATTGAATGCTGCAAAGAG	1620 bp
pMDC163_GUS-R	CCAGAGGTGCGGATTCACC	
E5F	TCAGCAAGTACATGAATATGTC	
HCC1E5-6R	GGACTCATCAAGTACATGACT	
E7R	TTACTTCCGGTACTGACGGA	
8409	ATATTGACCATCATACTCATTGC	
HCC1I6R	CTTCACCCACAACATGTTTAC	
Sco2E1F	GTCTTGTTCTGTCCTGCAAG	569 bp (cDNA)
Sco2E5R	CAAGTATGCGTGGAGATGAG	1.1 kb (genomic DNA)
UBCF	CAAAGAGGTACAGCGAGAG	381 bp (cDNA)
UBCR	CCTCTCACATCACCAGATC	1 kb (genomic DNA)
Sco2F	ATGCTTCCTTGTCGCCGTCT*	829 bp (cDNA)
Sco2R	ACTGTGAAACAGAAGCAACTTC*	
HCC2-BamHIF	CGGGGATCCATGCTTCCTTGTCGCC	1.5 kb (genomic DNA)
HCC2-SalIR	CGCGTCGACAGAAGCTCTTGTGAGAGC	
AHL1F	TAGTTAGTTACTTAAGCTCGGGC	

AHL2R	CAGAGCTGCAGCTGGATGGC	
mCherryR1	AAGCGCATGAACTCCTTGAT	
PP2AA3F	CCTGCGGTAATAACTGCATCT	142 bp (cDNA)
PP2AA3R	CTTCACTTAGCTCCACCAAGCA	
HCC2-RT-F	CGGATGTTGGACCTGAGCA	189 bp (cDNA)
HCC2-RT-R	TTGCACTTGCAGTCCCGGTTA	

*The attachment site sequences necessary for recombination were omitted. Only the gene-specific sequences are listed.
