# Supplementary figures and images for: Divergent functions of the Arabidopsis mitochondrial SCO proteins: HCC1 is essential for COX activity while HCC2 is involved in the UV-B stress response
Source: Front Plant Sci. 2014 Mar 25;5:87. doi: 10.3389/fpls.2014.00087 (PMC3971200; doi:10.3389/fpls.2014.00087)

**Supplementary Figure 1**

**
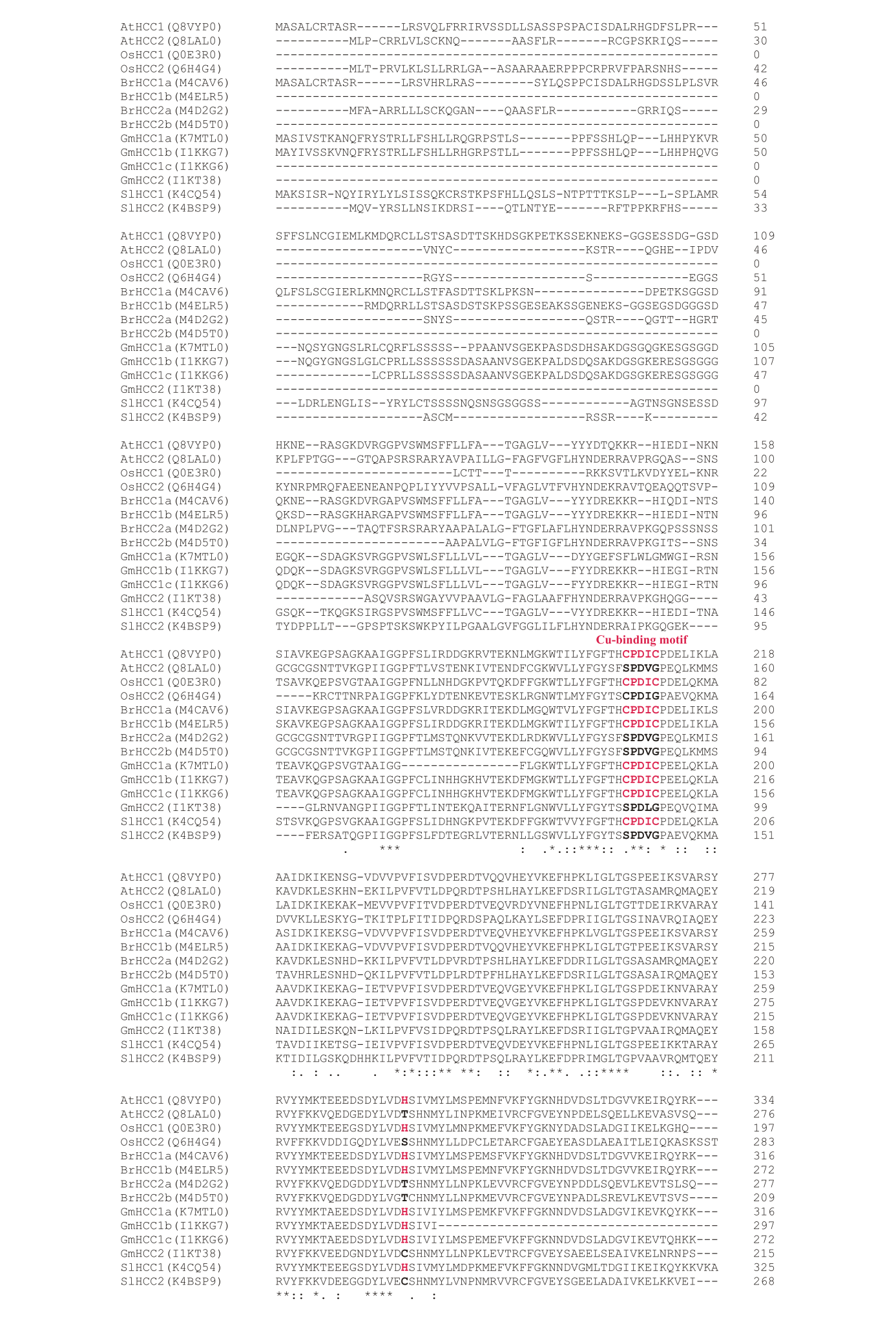
**

**Supplementary Figure 2**


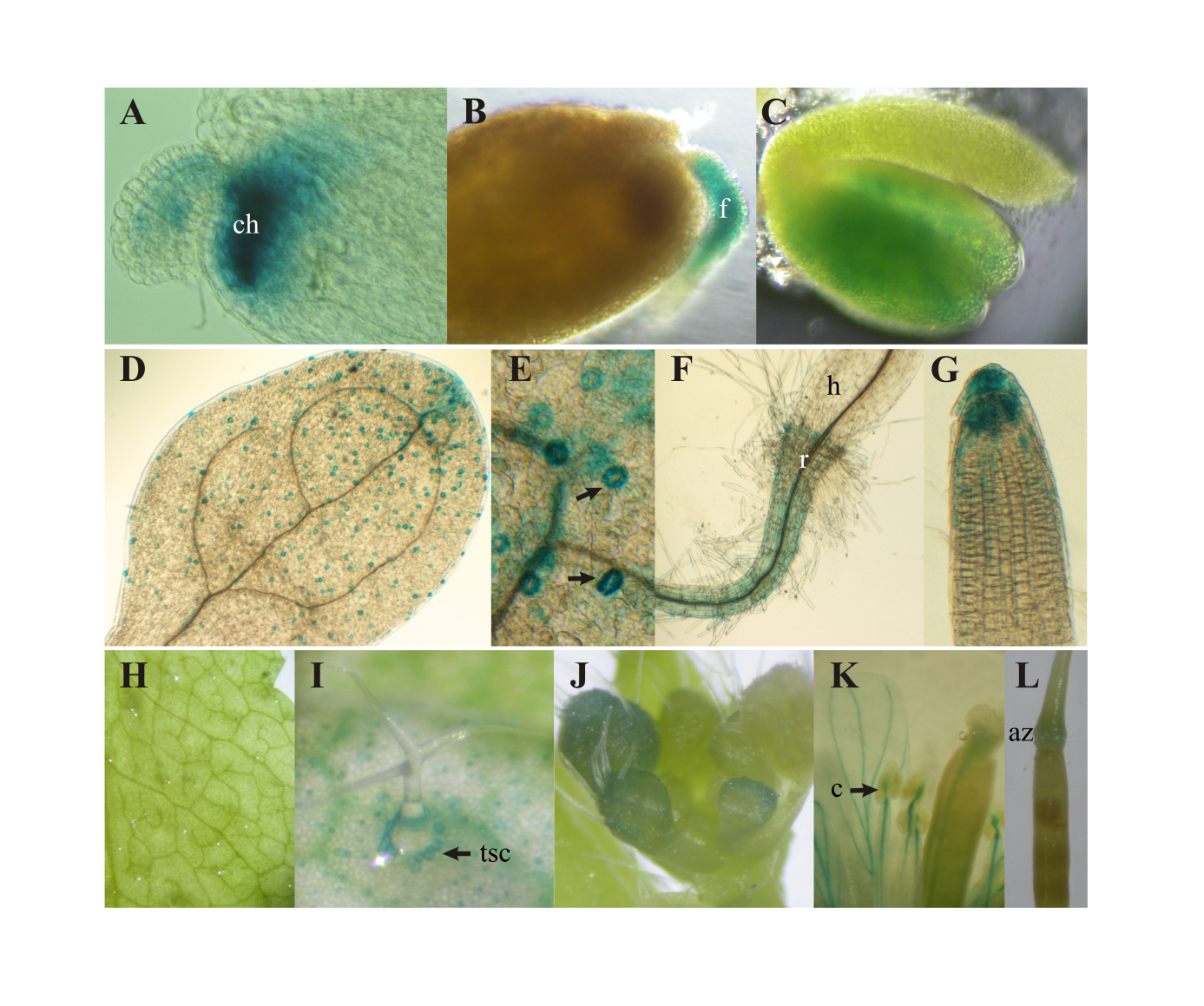


**Supplementary Figure 3**

**
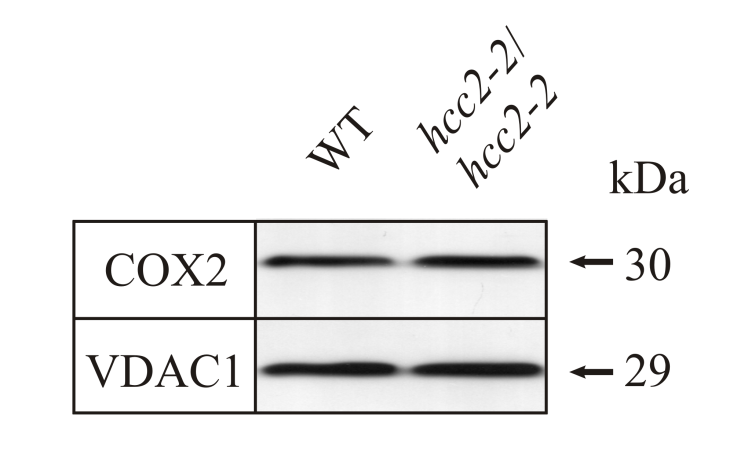
**

Supplement: Supplementary Figure 1 — Protein sequence alignment of SCO (HCC) proteins from plants: Arabidopsis thaliana (At), Oryza sativa subsp. japonica (Os), Brassica rapa subsp. pekinensis (Br), Glycine max (Gm), and Solanum lycopersicum (Sl). Protein sequences were retrieved from the UNIPROT database (UNIPROT numbers are given). The sequence alignment was performed using the ClustalO 1.2.0 alignment tool (Sievers et al., 2011). The consensus is quoted below the sequences (“*” conserved in all sequences, “.” partially conserved, “:” homology in all sequences). The amino acids involved in Cu-binding which includes the CxxxC motif as well as one histidine residue (Rentzsch et al., 1999; Nittis et al., 2001) are marked in red, if present. Proteins containing these conserved residues are titled “HCC1,” whereas those missing the motif are named “HCC2.” If more than one sequence in the UNIPROT database met this criterion, they were distinguished by different letters (a, b, c). [file DataSheet1.DOCX]
